# The creep model based on nonlinear Newton body under different temperature conditions

**DOI:** 10.1038/s41598-023-31983-0

**Published:** 2023-03-24

**Authors:** Lixin Zhang, Xiujie Wei, Yin Zhang

**Affiliations:** 1 College of Mining, Liaoning Technical University, Fuxin, 123000 China; 2 College of Mechanical and Engineering, Liaoning Technical University, Fuxin, 123000 China

**Keywords:** Energy science and technology, Materials science

## Abstract

Mastering the creep deformation characteristics of rock under different temperature conditions is of great significance for studying the long-term stability and deformation mechanism of geotechnical engineering. Based on the classical Burgers model, the creep model under different temperature conditions is established by introducing a nonlinear Newton body. The parameters of the creep model are identified and the influence law of different parameters on rock creep deformation is analyzed. The relationship between model parameters and temperature is quantitatively expressed. The results show that the newly established model can describe the characteristics of the rock in the decay creep stage and the constant creep stage, especially can quantitatively characterize the relationship between the strain and the time of the rock in the tertiary creep under different temperatures conditions. The model fitting curve is highly consistent with the test data, and the correlation coefficient *R*^2^ is above 0.98, which thoroughly verifies the accuracy and rationality of the model. It is found that when the temperature is constant, the creep increases with the increase of the shear modulus of the elastomer *G*_1_, the shear modulus of the viscoelastic body* G*_2_, and the viscosity coefficient of the viscous body *η*_1_ in the constant creep stage. The decay creep property of rock is more obvious with the increase of the viscosity coefficient *η*_2_, and the axial strain tends to a constant value. The achievement can be used to predict the deformation trend of geotechnical engineering with time under different temperature conditions and provide the theoretical basis for long-term stability analysis.

## Introduction

Exploring the creep characteristics of rock at different temperature conditions is an extremely important issue^1,2^ Many rock projects are subject to the dual effects of temperature changes and stress disturbances^3,4^, which will have a crucial impact on the mechanical properties of rocks and become a vital factor affecting the long-term stability of such rock mass projects^5–8^. Therefore, the study of rock creep characteristics under different temperature conditions has significant engineering application value.

Many researchers in the field of geotechnical engineering have done a lot of meaningful research work. From the perspective of micro-phase-only, Chen et al.^9^ discussed the influence mechanism of temperature on the microstructure characteristics of clay rock. Xue et al.^10^ considered the influence of deep salt rock formation temperature and volume stress on the rheological properties of salt rock. Based on the Drucker-Prager strength criterion, he introduced a generalized Hooke element considering plastic damage and established a constitutive model under triaxial compression. Niu et al.^11^ carried out high-temperature and high-pressure unsteady rheological tests on fine-grained granites to study its rheological. Yang et al.^12^ conducted uniaxial compression creep tests on sands under different temperature conditions and loading stresses to explore the influence of temperature on the strength characteristics and creep deformation laws of sands. Hu et al.^13^ used a multi-functional rock high-temperature triaxial testing machine to conduct uniaxial compression test on granite specimens, revealing the influence of temperature on the mechanical properties of granite. Liu et al.^14^ used confining pressure to simulate low-stress conditions, freezing red sandstone under different temperature conditions and confining pressures, tested its triaxial mechanical properties, and analyzed the effects of freezing confining pressure and temperature on mechanical properties. Zhang et al.^15^ carried out triaxial creep tests on gneisses granite at different temperature conditions and under different loading stress paths, and systematically analyzed the effects of temperature, confining pressure, and axial pressure on creep deformation characteristics, creep strength, and creep failure mode of gneisses granite. Although some relevant research results have been made in the rock creep model, the research on the theory of rock nonlinear constitutive model under different temperature conditions is still in infancy.

Based on the classical Burgers model, the creep model under different temperature conditions is established by introducing a nonlinear Newton body. Taking the triaxial creep test data of limestone as the research data, the rock creep characteristics under different temperature conditions and stress states are compared and analyzed, the mechanical mechanism of limestone deformation is discussed, and the parameters are identified by using *MATLAB* software.

## Analysis of limestone creep test data under different temperature conditions

**Figure 1:**
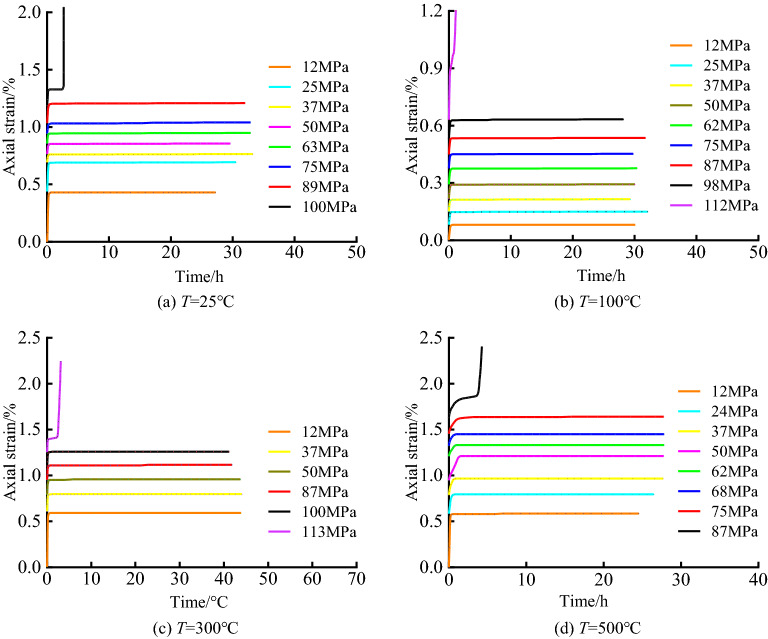
Triaxial compression creep curves of limestone under different temperature conditions.

Wu et al.^16^ used limestone with uniform texture along the Chenglan Railway to conduct triaxial creep tests on limestones at room temperature and experienced 100 ℃, 300 ℃, and 500 ℃ under the condition of σ^3^ = 10 MPa. Obtain the full-process curve of creep strain–time of limestone under triaxial compression under different temperature conditions as shown in Fig. 1.

As shown in Fig. 1 that when the loading stress level is less than the rock yield strength, in the early stage of the test, the rock sample appears instantaneous elastic strain under stress loading, and the loading time is short in this stage. Then, the rock sample enters the decay creep stage, and the slope of the creep curve decreases gradually in this stage, showing obvious nonlinear characteristics, and the attenuation process and characteristics are closely related to the rock stress and temperature when the rock enters the constant creep stage. With the creep test, the rock creep strain tends to be stable gradually with the increase of time, and the creep has reached a stable state. When the loading stress level is greater than the rock yield strength, the rock quickly enters the tertiary creep. In this stage, the strain and strain rate increase significantly, and the nonlinear characteristics of the strain curve are obvious.

## Establishment of the creep model under different temperature conditions

**Figure 2:**
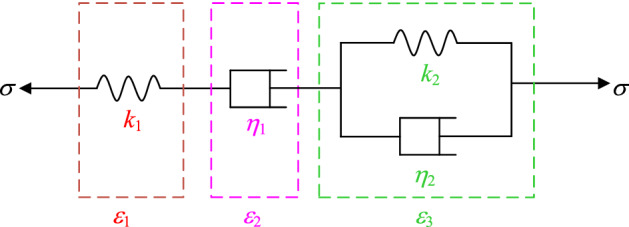
Classic Burgers model.

**Figure 3:**
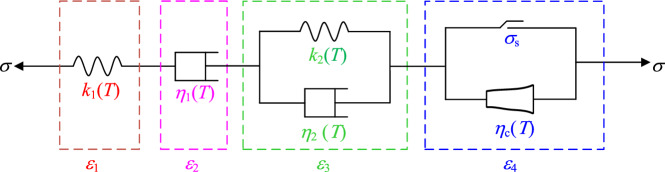
Nonlinear creep model.

The basic creep models of rock include the classic Burgers model^17^, the classic Nishihara model, the Maxwell model, and the Kelvin model^18^. However, these models cannot consider the creep deformation law of the third stage- tertiary creep of rock and cannot effectively consider the creep characteristics of the rock under different temperature conditions^19,20^ and JG et al.^21^). The actual environment of rock rheology is very complex, and the analysis of its rheological law can be carried out in a variety of combinations. In this regard, based on the classic Burgers model (as shown in Fig. 2), the author introduced a nonlinear Newton body, and constructed a constitutive creep model under different temperature conditions, as shown in Fig. 3.

### The creep equation of components

#### The creep equation of the Burgers model

It can be seen from Fig. 2 that the classical Burgers model is composed of a spring, a clay pot, and a Kelvin body in series. It is a typical four-element viscoelastic rheological model, which can better describe the decay creep stage and constant creep stage deformation laws of rock and rock mass engineering^22^. However, this model cannot effectively describe the law characteristics of tertiary creep deformation of rock (Cai and Cao.^23^).

In a one-dimensional stress state, the creep equation of Burgers model is as follows (Wang and Sheng^24^):

1 $$\varepsilon = \frac{\sigma }{{k_{1} }} + \frac{\sigma }{{\eta_{1} }}t + \frac{\sigma }{{k_{2} }}\left[ {1 - \exp ( - \frac{{k_{2} }}{{\eta_{2} }}t)} \right],$$

where *ε* is the strain, %; *σ* is the stress, MPa; *k*_1_ is the elastic modulus of the elastomer, MPa; *k*_2_ is the elastic modulus of the viscoelastic body, MPa; *η*_1_ is the viscosity coefficient of the viscous body, MPa·h; and *η*_2_ is the viscosity coefficient of the viscoelastic body, MPa·h.

In a three-dimensional stress state, the creep equation of Burgers model is as follows^25^:

2 $$\varepsilon_{ij} = \frac{{S_{ij} }}{{2G_{1} }} + \frac{{\sigma_{m} \delta_{ij} }}{3K} + \frac{{S_{ij} }}{{\eta_{1} }}t + \frac{{S_{ij} }}{{G_{2} }}\left[ {1 - \exp ( - \frac{{G_{2} }}{{\eta_{2} }}t)} \right],$$

where *ε*_*ij*_ is the strain, %; *S*_*ij*_ is deviation stress tensor, MPa;* G*_1_ is the shear modulus of the elastomer, MPa;* σ*_m_ is average stress, MPa; *δ*_*ij*_ is Kronecker tensor, MPa; *K* is the bulk modulus of the elastomer, MPa; *G*_2_ is the shear modulus of the viscoelastic body, MPa.

#### The creep equation of elastomer

The elastic modulus of the elastomer is not affected by time, so the elastic strain of limestone under different temperature conditions^26^:

3 $$\varepsilon_{1} = \frac{\sigma }{{k_{1} (T)}},$$

where *ε*_1_ is the strain of the elastomer, %; *k*_1_(*T*) is the elastic modulus of the elastomer under different temperature conditions, MPa.

The creep equation of the elastomer under three-dimensional stress can be written as follows^27^:

4 $$\varepsilon_{ij}^{1} = \frac{3(1 - 2v)}{{2k_{1} (T)}}S_{ij} + \frac{2(1 + v)}{{3k_{1} (T)}}\sigma_{m} \delta_{ij} = \frac{{S_{ij} }}{{2G_{1} (T)}} + \frac{{\sigma_{m} \delta_{ij} }}{3K},$$

where *v* is the Poisson’s ratio of rock; *G*_1_(*T*) is the shear modulus of the elastomer under different temperature conditions, MPa.

And the deviatoric stress tensor in the principal stress space can be expressed:

5 $$S_{ij} { = }\sigma_{ij} - \sigma_{m} \delta_{ij} .$$

#### The creep equation of the viscous body

The viscous body is also called the damping element. Its constitutive equation is^28^:

6 $$\sigma = \eta_{1} (T)\frac{{d\varepsilon_{2} }}{dt},$$

where *η*_1_(*T*) is the viscosity coefficient of the viscous body under different temperature conditions, MPa·h.

By integral transformation, the one-dimensional creep equation of viscous body is:

7 $$\varepsilon_{2} = \frac{\sigma }{{\eta_{1} (T)}}t.$$

The creep equation of the viscous body under a three-dimensional stress state is as follows:

8 $$\varepsilon_{ij}^{2} = \frac{{\sigma_{1} - \sigma_{3} }}{{\eta_{1} (T)}}t.$$

#### The creep equation of the viscoelastic body

The viscoelastic body is also called the Kelvin model. The classical Kelvin model can accurately describe the nonlinear characteristics of the creep curve. The constitutive equation of the viscoelastic body is as follows:

9 $$\sigma = \eta_{2} (T)\frac{{d\varepsilon_{3} }}{dt} + k_{2} (T)\varepsilon_{3} ,$$

where *ε*_3_ is the strain of the viscoelastic body, %;* k*_2_(*T*) is the elastic modulus of the viscoelastic body, MPa; *η*_2_(*T*) is the viscosity coefficient of the viscoelastic body under different temperature conditions, MPa·h.

A one-dimensional creep equation of the viscoelastic body under different temperature conditions is established as follows:

10 $$\varepsilon_{3} = \frac{\sigma }{{k_{2} (T)}}\left[ {1 - \exp ( - \frac{{k_{2} (T)}}{{\eta_{2} (T)}}t)} \right].$$

Extending to three-dimensional space, the three-dimensional creep equation of the viscoelastic body under different temperature conditions can be obtained as:

11 $$\varepsilon_{ij}^{3} = \frac{{S_{ij} }}{{2G_{2} (T)}}\left[ {1 - \exp ( - \frac{{G_{2} (T)}}{{\eta_{{2}} (T)}}t)} \right],$$

where *G*_2_(*T*) is the shear modulus of the viscoelastic body under different temperature conditions.

#### The creep equation of the nonlinear Newton body

**Figure 4:**
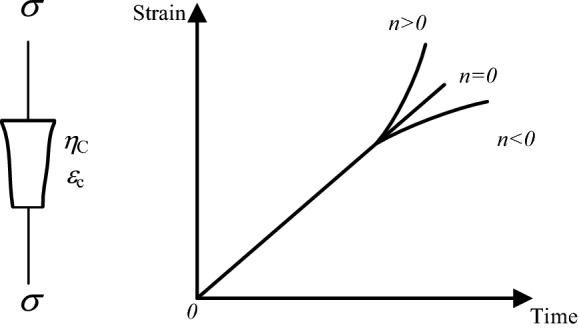
Nonlinear Newton body and creep curve.

When the stress is greater than the yield strength (*σ* > *σ*_s_), the rock enters the tertiary creep, and the amount of creep deformation and creep rate increase nonlinearly with time. The research introduces a nonlinear Newton body (as shown in Fig. 4)^29,30^, which is composed of a variable cross-section Newton damper, and its initial region length is *ε*_c_. When *ε* < *ε*_c_, the linear Newtonian has similar characteristics, that is $\dot{\varepsilon } = \frac{\sigma }{{\eta_{c} }}$; when *ε* ≥ *ε*_c_, $\dot{\varepsilon }$ is the *n*-th power function relationship of time, that is $\dot{\varepsilon } = \frac{\sigma }{{\eta_{c} }}At^{n}$^31,32^.

And as can be seen from Fig. 4, When *n* > 0, the nonlinear Newton body reflects the acceleration process of rock creep. When *n* < 0, the creep strain rate of the nonlinear Newton body decreases with the increase of creep time, which can describe the attenuation process of rock creep. When *n* = 0, the nonlinear Newton body appears as a linear viscous body.

Therefore, the one-dimensional creep equation of the nonlinear Newton body is

12 $$\varepsilon_{4} = \frac{{A(\sigma - \sigma_{s} )}}{{(n + 1)\eta_{c} (T)}}(t - t_{a} )^{n + 1} ,$$

where *A* and *n* are undetermined constants; *σ*_s_ is the yield strength, MPa; *t*_a_ is the moment when tertiary creep is entered, h; *η*_c_(*T*) is the viscosity coefficient of the nonlinear Newton body under different temperature conditions, MPa·h.

The three-dimensional creep equation of the nonlinear Newton body is

13 $$\varepsilon_{ij}^{4} = \frac{{A(\sigma_{1} - \sigma_{3} - \sigma_{s} )}}{{(n + 1)\eta_{c} (T)}}(t - t_{a} )^{n + 1} .$$

### Establishment of the creep model based on nonlinear Newton body

It is found that when *σ* < *σ*_s_, the model degenerates into a Burgers model under different temperature conditions, which can describe the elastic-stick-viscoelastic rheological properties of rock materials; when *σ* > *σ*_s_, the rock will appear tertiary creep. According to the superposition principle of components^33,34^, the creep equation of the creep model based on nonlinear Newton body can be obtained as follows:

14 $$\left\{ {\begin{array}{*{20}c} {\varepsilon = \varepsilon_{1} + \varepsilon_{2} + \varepsilon_{3} + \varepsilon_{4} } \\ {\varepsilon_{ij} = \varepsilon_{ij}^{1} + \varepsilon_{ij}^{2} + \varepsilon_{ij}^{3} + \varepsilon_{ij}^{4} } \\ \end{array} } \right..$$

In the conventional triaxial compression creep test, the second principal stress *σ*_2_ = *σ*_3_, then:

15 $$\left\{ {\begin{array}{*{20}c} {\sigma_{m} = \frac{1}{3}(\sigma_{1} + \sigma_{{2}} + \sigma_{3} ) = \frac{1}{3}(\sigma_{1} + 2\sigma_{3} )} \\ {S_{{{11}}} = \sigma_{1} - \sigma_{m} = \frac{2}{3}(\sigma_{1} - \sigma_{3} )} \\ \end{array} } \right.,$$

where *σ*_1_ is axial compression, MPa; *σ*_2_ and *σ*_3_ are confinement pressure, MPa; *S*_11_ is axial deviatoric stress tensor, MPa.

Based on Eqs. (3), (7), (10), (12) and (14), the one-dimensional creep equation of the creep model based on nonlinear Newton body under different temperature conditions can be obtained as:

16 $$\varepsilon = \left\{ {\begin{array}{*{20}c} {\frac{\sigma }{{k_{1} (T)}} + \frac{\sigma }{{\eta_{1} (T)}}t + \frac{\sigma }{{k_{2} (T)}}\left[ {1 - \exp ( - \frac{{k_{2} (T)}}{{\eta_{2} (T)}}t)} \right]} & {\sigma < \sigma_{s} } \\ {\frac{\sigma }{{k_{1} (T)}} + \frac{\sigma }{{\eta_{1} (T)}}t + \frac{\sigma }{{k_{2} (T)}}\left[ {1 - \exp ( - \frac{{k_{2} (T)}}{{\eta_{2} (T)}}t)} \right] + \frac{{A(\sigma - \sigma_{S} )}}{{(n + 1)\eta_{c} (T)}}(t - t_{a} )^{n + 1} } & {\sigma > \sigma_{s} } \\ \end{array} } \right..$$

Based on Eqs. (4), (8), (11), (13), (15) and (14), the three-dimensional creep equation of the creep model based on nonlinear Newton body under different temperature conditions can be obtained as:

17 $$\varepsilon_{ij} = \left\{ {\begin{array}{*{20}c} {\frac{{\sigma_{1} - \sigma_{3} }}{{3G_{1} (T)}} + \frac{{\sigma_{1} + 2\sigma_{3} }}{9K} + \frac{{2(\sigma_{1} - \sigma_{3} )}}{{3\eta_{1} (T)}}t + \frac{{\sigma_{1} - \sigma_{3} }}{{3G_{2} (T)}}\left[ {1 - \exp ( - \frac{{G_{2} (T)}}{{\eta_{2} (T)}}t)} \right]} & {\sigma < \sigma_{s} } \\ {\frac{{\sigma_{1} - \sigma_{3} }}{{3G_{1} (T)}} + \frac{{\sigma_{1} + 2\sigma_{3} }}{9K} + \frac{{2(\sigma_{1} - \sigma_{3} )}}{{3\eta_{1} (T)}}t + \frac{{\sigma_{1} - \sigma_{3} }}{{3G_{2} (T)}}\left[ {1 - \exp ( - \frac{{G_{2} (T)}}{{\eta_{2} (T)}}t)} \right] + \frac{{A(\sigma_{1} - \sigma_{3} - \sigma_{S} )}}{{(n + 1)\eta_{c} (T)}}(t - t_{a} )^{n + 1} } & {\sigma > \sigma_{s} } \\ \end{array} } \right..$$

## Model parameter identification and validation

**Figure 5:**
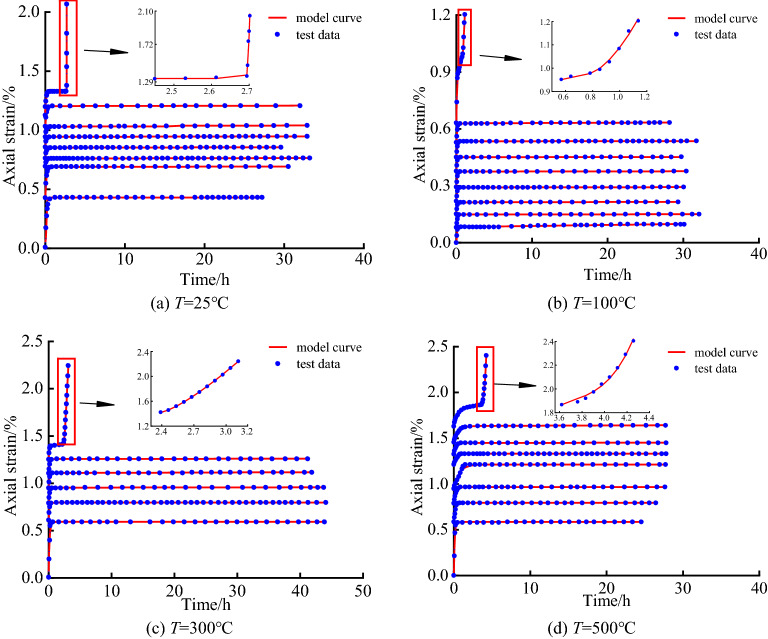
Comparative analysis of model curve and test data under different temperature conditions.

To verify the correctness and rationality of the creep model based on nonlinear Newton body under different temperature conditions, the parameter values of the creep model are determined based on the limestone test data in this study. Rock creep model parameters are usually determined by two methods, one is graphic fitting method^35^, this method is based on the relationship between the morphological meaning of rock creep curve and model parameters, and then through the data to fit the corresponding parameters, another method is optimization analysis^15,18^, which usually uses regression analysis and least square method to determine the model parameters. Compared these two methods, the second method has higher accuracy, simple method and stronger applicability. Therefore, the least square method is used to fit the model parameters, and the results of model parameter identification are shown in the Table 1. The model curve was compared with the experimental data under different temperature conditions as shown in Fig. 5.

**Table 1:** Model parameter identification results.

Temp/°C	Deviatoric stress/MPa	*G*_1_(*T*)/MPa	*η*_1_(*T*)/MPa·h	*G*_2_(*T*)/MPa	*η*_2_(*T*)/MPa·h	*A*	*n*	*η*_c_(*T*)/MPa·h	*R*^2^
25 °C	12	230.7183	11,566.313	11.502	1.352				0.9803
	25	72.0337	819,059.24	31.618	4.135				0.9895
	37	60.3613	159,171.81	183.422	20.918				0.9983
	50	70.4842	225,921.59	187.032	23.048				0.9972
	63	76.7957	468,428.62	239.113	23.681				0.9990
	75	81.4769	109,576.96	286.969	35.386				0.9963
	89	86.9330	581,289.57	170.936	23.271				0.9963
	100	88.5508	409,283.8	165.636	15.186	16.025	103.992	1.457	0.9933
100 °C	12	1194.86029	104,447.12	18.840	1.228				0.9929
	25	392.7862	425,876.21	117.920	15.529				0.9938
	37	278.7673	279,967.46	195.720	20.259				0.9976
	50	248.9522	482,975.53	220.867	25.719				0.9959
	62	222.2164	622,522.41	247.675	31.737				0.9963
	75	203.9905	818,836.92	336.684	36.664				0.9964
	87	196.2913	904,960.29	333.627	40.323				0.9958
	98	186.5177	353,380.85	313.185	38.910				0.9936
	112	177.3852	467.742	144.179	13.870	9.968	6.602	6.175	0.9923
300 °C	12	175.3056	53,485.456	5.079	0.507				0.9920
	37	69.1009	35,331.649	64.455	6.045				0.9977
	50	68.1851	320,690	100.712	13.774				0.9976
	87	92.6334	237,071.16	180.16	20.650				0.9969
	100	90.8131	1,638,116.9	208.745	28.558				0.9932
	113	89.3547	6329.094	259.312	24.160	10.622	3.118	7.14	0.9952
500 °C	12	125.3327	563,059.57	8.8371	1.172				0.9906
	24	51.9089	614,259.51	36.565	7.252				0.9958
	37	53.1910	819,059.32	68.350	17.538				0.9987
	50	57.1937	1,228,659.6	61.310	42.249				0.9905
	62	53.6134	1,049,657.3	169.946	62.815				0.9953
	68	53.0342	1,228,518.7	184.053	49.934				0.9984
	75	52.5812	119,779.18	144.827	81.099				0.9987
	87	53.6213	2916.331	165.942	75.860	0.121	67.207	3.945	0.9961

It can be seen from Fig. 5 that there are certain errors between the model curve and the test data at different temperature conditions. The test data are scattered on both sides of the model curve, which is caused by the inevitable difference between the samples, which also shows the rationality and correctness of the model construction. The correlation coefficient of model fitting test data are all greater than 0.98, indicating that the creep model based on nonlinear Newton body proposed in the research can accurately reflect the whole process of creep deformation of rock under different temperature conditions. Especially for the simulation effect of the tertiary creep, the nonlinear variation characteristics of tertiary creep can be well captured, which is in good agreement with the test data.

Based on the above fitting results, this model can better reflect the typical three-stage creep characteristics of rock and is in good agreement with the test data, which verifies the effectiveness of the model and identification method. Therefore, it is reasonable and feasible to use the creep model based on nonlinear Newton body established in the research to describe the creep characteristics of rock under different temperature conditions. The research results can be used as an important reference for the long-term stability analysis of rock mass engineering under severe temperature change or high-temperature environments.

**Figure 6:**
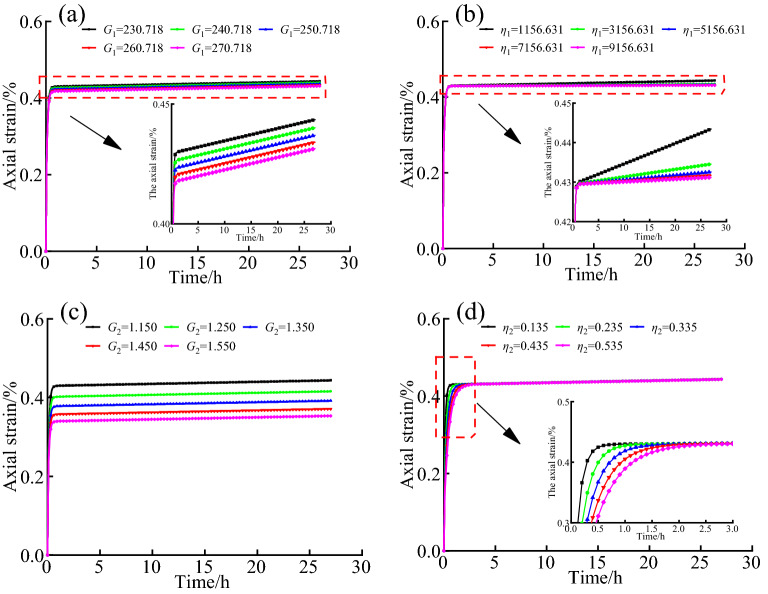
Creep curves of rock with different *G*_1_, *η*_1_, *G*_2_ and *η*_2_ (**a**) *G*_1_, (**b**)* η*_1_, (**c**)* G*_2_, (**d**) *η*_2_.

To explore the influence of model parameters on rock creep deformation, the following Fig. 6 shows the influence of different values of parameters *G*_1_,* η*_1_, *G*_2_, and* η*_2_ on rock deformation.

It can be seen from Fig. 6a and Fig. 6c, the creep amount at the end of rock decay creep stage decreases with the increase of *G*_1_ and *G*_2_ values, and the creep amount of rock decreases with the increase of shear modulus, but the creep rate is the same after entering the constant creep stage, and the sensitivity of *G*_2_ is stronger than that of *G*_1_. In Fig. 6b, the larger the viscosity coefficient *η*_1_ is, the smaller the creep rate of rock at the constant creep stage is. In Fig. 6d, with the increase of viscosity coefficient *η*_2_, the decay creep property of rock is more obvious, and the slower it enters the constant creep stage, but the final deformation is the same (function property of Eq. (9)). The figures in Fig. 6 show obvious decay creep stage and constant creep stage, indicating that the defined functional relationship under different temperature conditions is feasible to simulate the decay creep deformation and constant creep deformation of rock.

**Figure7:**
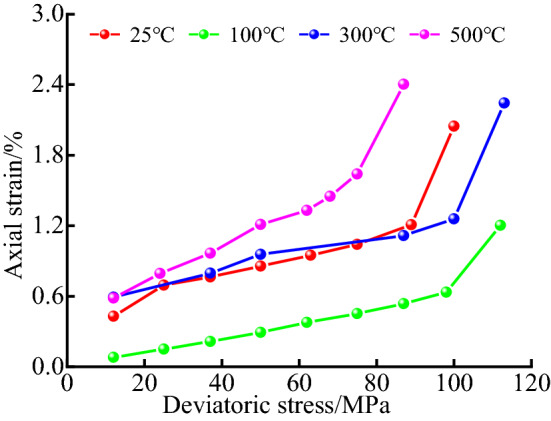
Curve of axial strain change against deviatoric stress under different temperature conditions.

Figure 7 shows the curves of axial strain change against deviatoric stress at different temperatures. The analysis shows that at the end of the tertiary creep stage of rock, the final creep amount at different temperatures is 2.046% at 25 °C, 1.203% at 100 °C, 2.244% at 300 °C, and 2.405% at 500 °C, respectively. According to the regular analysis of temperature on rock deformation, this result is obviously incorrect. Therefore, it is necessary to explain this result through the wave velocity test in the original literature^16^ of the data.

Table 2 is the longitudinal wave velocity results of limestone samples before and after heating at four temperatures. Table 2 is analyzed and explained as follows. At room temperature, the wave velocity of the rock has almost no change, indicating that there is no physical and chemical change in the rock at room temperature. The increase of wave velocity after heating at 100 °C is due to the loss of free water in the rock and the thermal expansion of internal mineral particles, which makes the contact between particles denser, and the temperature has an obvious hardening effect on limestone. Therefore, the creep of rock samples at 100 °C is lower than that at room temperature. When the heating temperature rises to 300 °C, the creep amount of the rock sample recovers to the same order of magnitude as that of the room temperature state, even higher than that of the room temperature state. The reason is that under the condition of high temperature, cracks appear between particles and particles in the rock, and pores appear along with the discharge of water vapor. Under the action of external force, the friction of particles gradually fills the pores, and the macroscopic performance is that the creep is worth increasing. When the rock sample is heated to 500 °C, the wave velocity decreases compared with that before heating. This is because the thermal expansion cracks are generated between the components on the physical layer surface due to the differences in the mineral composition and thermal expansion coefficient inside the rock. With the reaction of chemical composition, the internal gap further increases, and the macroscopic performance is the increase of rock creep. However, the rock properties have been attenuated at the micro level, and the rock will be destroyed quickly under external force. Therefore, the creep of rock samples heated at 500 °C is larger than that of the first three temperatures, but the long-term strength is smaller than that of the first three temperatures.

**Table 2:** Wave velocity changes of limestone before and after heating at different temperatures.

Limestone state	Wave velocity (m/s) of limestone at different temperatures (°C)			
	25 °C	100 °C	300 °C	500 °C
Before heating	6301	5971	6352	6081
After heating	6301	6854	6691	5084

## Next step of research

According to statistics, the number of open-pit mines in Inner Mongolia, Xinjiang, Gansu, and other Northwest China in 2021 has reached 83.39% of the national number of open-pit mines. Rock mass engineering has long experienced the huge temperature difference change of seasonal alternation and day-night cycle, resulting in different degrees of freeze–thaw disasters such as frost heaving cracking, freeze–thaw collapse, and slope instability. Therefore, this paper will continue to study the influence of low temperature on rock creep properties and the establishment of a constitutive model.

## Conclusion

Based on the classical Burgers model, the creep model under different temperature conditions is established by introducing a nonlinear Newton body. The parameters of the creep model are identified and the influence law of different parameters on rock creep deformation is analyzed. The relationship between model parameters and temperature is quantitatively expressed. The following main conclusions are drawn:

1. The newly established creep model can describe the whole-process creep characteristics of rock under different temperature conditions. It overcomes the deficiency that the classical Burgers model cannot describe the tertiary creep.
2. The correlation coefficients *R*^2^ of the theoretical analysis and the test data is greater than 0.98, which fully verifies the rationality and accuracy of the newly established creep model.
3. When the temperature is constant, the creep increases with the increase of the shear modulus of the elastomer *G*_1_, the shear modulus of the viscoelastic body *G*_2_, and the viscosity coefficient of the viscous body *η*_1_ in the constant creep stage. The decay creep property of rock is more obvious with the increase of the viscosity coefficient *η*_2_, and the axial strain tends to a constant value.

## Data Availability

The data used to support the findings of this study are available from the corresponding author upon request.
